# Polymorphic variants in genes related to stress coping are associated with the awake bruxism

**DOI:** 10.1186/s12903-021-01844-1

**Published:** 2021-10-05

**Authors:** Zofia Maciejewska-Szaniec, Marta Kaczmarek-Ryś, Szymon Hryhorowicz, Agnieszka Przystańska, Tomasz Gredes, Barbara Maciejewska, Justyna Hoppe-Gołębiewska, Ryszard Słomski, Andrzej Pławski, Agata Czajka-Jakubowska

**Affiliations:** 1 Department of Temporomandibular Disorders, Poznan University of Medical Sciences, Poznan, Poland; 2 Institute of Human Genetics, Polish Academy of Sciences, Poznan, Poland; 3 Department of Orthodontics Medical Faculty, Carl Gustav Carus TU, Dresden, Germany; 4 Department and Clinic of Phoniatrics and Audiology, Poznan University of Medical Sciences, Poznan, Poland

**Keywords:** Awake bruxism, Susceptibility, Stress coping, *BDNF* gene, *NTRK2* gene, Association analysis

## Abstract

**Background:**

Chronic stress is one of the leading predisposing factors in bruxism aetiology, but the influence of genetic factors is also suggested. We aimed to study whether sequence variants in genes involved in stress regulation pathways: *NTRK2* and *BDNF*, may be associated with awake bruxism susceptibility, clinical presentation, and patients’ perceived stress level.

**Methods:**

The study group included 104 patients with probable awake bruxism and 191 population controls. Patients underwent dental examination concerning the symptoms of bruxism and psychological testing. Genotyping was performed using HRMA and sequencing. Statistical analyses were conducted, and *P *values below 0.05 were considered statistically significant.

**Results:**

We observed a positive correlation of measured stress level and pathological teeth attrition in the anterior segment (r = 0.45, *P* < 0.001), enamel attritions (r = 0.44, *P* < 0.001), tongue impressions (r = 0.50, *P* < 0.001) and posterior teeth attrition (r = 0.27, *P* = 0.005). Moreover, the c.196A variant (p.66Met) of the *BDNF* gene and c.1397-31392G allele of the *NTRK2* gene were present with elevated frequency, comparing to controls.

**Conclusions:**

This study hence the thesis that perceived stress level is a substantial contributing factor to awake bruxism occurrence and its clinical manifestations. Moreover, sequence variants in genes related to stress coping may be correlated with awake bruxism’s susceptibility via elevated perceived stress level.

## Background

The definition of bruxism has been under debate for some time. However, since 2013 it is defined as a repetitive jaw-muscle activity characterized by teeth grinding and/or clenching and/or bracing or thrusting the mandible. Bruxism may have two manifestations: during wakefulness (awake bruxism, AB) and sleep (sleep bruxism, SB). In 2018, it was argued that AB is a masticatory-muscle activity that occurs during wakefulness. It is characterized by repetitive or sustained tooth contact and/or bracing or thrusting of the mandible. SB, occurring during sleep, might be rhythmic (phasic) or non-rhythmic (tonic). Neither of the bruxism forms should be considered a movement or sleep disorder, as in most individuals, it is not an isolated condition but a symptom in the manifestation of other disorders. The grading system proposed by Lobbezoo et al. suggests that diagnosis of possible sleep/awake bruxism could be made on a positive patient’s self-report only. When clinical findings are positive independently of positive self-report, sleep/awake bruxism may be recognized as probable, and to establishing the diagnosis of definite sleep/awake bruxism, a positive instrumental examination is required, regardless of a positive self-report and/ or positive clinical findings [1–3].

The development of bruxism is influenced by several factors: the intensity and duration of etiological factors activity, their co-occurrence, and the ability of stomatognathic system structures to accept changing conditions that arise with age but on the top of the leading predisposing factors of the aetiology of bruxism is civilization chronic stress [4, 5].

The patient’s personality type and the ability to dealing with chronic stress is no less critical. The psychoemotional variable is essential to increasing the masticatory organ’s muscular tone, including the strongest rumen muscle. Increased muscle tension, following the principle of “vicious circle,” being the effect becomes the cause of the symptoms escalating. Initially, the body adapts to a stressful situation. However, after crossing the threshold, changes in tissues and dysfunctions accompanying intense pain symptoms occur. In the last phase, alterations in the stomatognathic system structures become irreversible [4–6].

The current state of medical knowledge does not clearly identify the cause and bruxism mechanism. However, the aetiology of bruxism is undoubtfully multifactorial and reflects from the accumulation of several causatives and contributing factors. Several papers also suggested the significant influence of genetic factors [7–14]. Studies on the genetic background of the masticatory organ motion system’s dysfunction strongly suggest their multigene conditioning. The genome-wide association studies (GWAS), including large study groups, identified numerous genes that may be associated with a genetic predisposition to disorders of the stomatognathic system [15]. Notably, several signalling pathways involved in neuronal plasticity have been demonstrated to be impaired in patients with mood disorders or animal models exposed to stress [16–18]. Other studies have also indicated that the expression of critical molecules involved in regulating synaptic plasticity, such as brain-derived neurotrophic factor (neurotrophin, BDNF), is reduced in response to stress [18].

Aizawa et al. showed that the *BDNF* and *NTRK2* gene (encoding the neurotrophin receptor) polymorphisms modulate individual responses to stress and stress coping styles [19]. In our opinion, this observation may have a strong connection with the occurrence of bruxism.

In this study, we aimed to analyze sequence variants in *BDNF* and *NTRK2* genes, which are known as involved in stress regulation pathways in patients with probable AB, to determine their association with AB susceptibility.

## Methods

### Study subjects

The study group included 104 non-related, adult Caucasian patients (39 males and 65 females) examined and diagnosed with probable AB at the Department of Temporomandibular Disorders, Division of Prosthodontics, Poznan University of Medical Sciences. Patients were prospectively enrolled in the study.

The controls were 191 unrelated adult individuals from the Polish population (96 males and 95 females from the Great Poland region) attending paternity testing. All individuals gave their written consent to genetic testing.

### Dental examination

Clinical evaluation was carried out using a questionnaire based on the Oral Behaviours Checklist (OBC) [20], containing the questions: ‘do you clench your teeth during waking hours?’, ‘do you press, touch, or hold your teeth together with other than while eating (that is, contact between upper and lower teeth)?’, and ‘do you hold, tighten, or tense muscles without clenching or bringing teeth together?’ (responses on a 5-point Likert scale: ‘none of the time’ was scored as 0, ‘all of the time’ was scored as 4). When participants answered more than or equal to ‘sometimes (score 2)’, possible awake bruxism was recorded as being present. The clinical examination contains: the occurrence of hypertrophy and tenderness of the masticatory muscles, impressions on the lateral surface of the tongue shaft, indentations in cheek and/or lip, damage to the dental hard tissues (intrinsic mechanical tooth wear, crumbling teeth, cracked teeth or tooth mobility), repetitive failures of restorative work/prosthodontic constructions with or without positive self-reporting.

The inclusion criteria were: age above 18 years, positive history of tooth clenching when awake confirmed by the patient during the interview; score minimum 2 in three questions from the OBC questionnaire listed above, and at least two of the following signs/symptoms: discomfort/pain during palpation in one masseter or temporalis muscles site per side lasting from 3 months, hypertrophy of masseters, impressions on the lateral surface of the tongue shaft, indentations in cheek or lip, damage to the dental hard tissues—tooth wear (using Martin’s tooth wear index—the 3rd class was accepted), repetitive failures of restorative work/prosthodontic constructions.

The exclusion criteria from the study were as follows: age over 40 years, secondary bruxism induced by systemic diseases, e.g., Parkinson’s, neurological disorders and/or neuropathic pain (epilepsy, vagus nerve neurosis), medicines that can significantly affect the functioning of the nervous and muscular systems; severe mental disorders, compensated systemic diseases (cardiovascular diseases, diabetes mellitus), primary congenital SS changes cancer, implanted pacemaker, pregnancy, lactation, intellectual disability, injuries and operations in the craniofacial area in the past.

### Psychological examination

The Perceived Stress Scale (PSS-10) psychological test by Cohen et al. [21] was carried out to measure the level of perceived stress (PSS-10 is distributed in Poland by the Psychological Test Laboratory of the Polish Psychological Association (https://www.practest.com.pl/npsr-narzędzia-pomiaru-stresu-i-radzenia-sobie-ze-stresem). It contains ten questions about different subjective feelings related to personal problems, events, behaviours, and ways of dealing with them. The respondent gave his answers, entering the number (0—never, 1—rarely, 2—sometimes, 3—fairly often, 4—very often). The overall result of the scale is the sum of all points theoretically distributed from 0 to 40: the higher score, the greater severity of perceived stress. General indicator after transformation on standardized units is subject to interpretation according to the characteristics of the sten scale. Results between 1 and 4 stens have been interpreted as low and within 7–10 stens as high. Results within 5 and 6 stens have been interpreted as average [21, 22].

### DNA extraction and genotyping

DNA was extracted from whole blood leukocytes using guanidine isothiocyanate and phenol–chloroform [23]. Isolates were dissolved in 1xTE buffer and stored at − 20 °C until use. The genotyping of the sequence variants: c.196G > A (p.Val66Met, rs6265) in *BDNF* and c.1397-31392G > A (rs1867283) in *NTRK2* gene, was performed by using high resolution melting (HRM) analysis and Sanger sequencing. The following primers were used to amplification of gene regions under interests: sense 5′-TGAGAGCAACGTAAGGCATTT and antisense 5′-ACGAGGGCCTCCTTATGTTT (Tm = 60 °C) giving a PCR product of 249 bp, for rs6265, and sense 5′-AAACATCCGAGGACAAGGTG and antisense 5′-AGAAGAGGAGGCTCCAAAGG (Tm = 60 °C) giving a PCR product of 160 bp, for rs1867283, respectively. The reaction was carried out on the Rotor-Gene® (Qiagen) using a commercial set of Type-it HRM PCR Kit (Qiagen) reagents. Sanger sequencing was used for the sequence variants identification in samples with different melting temperatures, using the commercial BigDye™ Terminator v3.1 Cycle Sequencing Kit and the ABI PRISM 3100 instrument (Applied Biosystems) according to manufacturer procedures.

### Statistical analysis

Categorical data were expressed as counts and percentages and were compared using a chi-squared test. The correlation coefficient has been used to measure the strength of an association between two variables, where the r value = 1 means a perfect positive correlation and = − 1 means a perfect negative correlation. The point-biserial correlation coefficient calculator has been used in exceptional circumstances when one of the variables was dichotomous. The Kolmogorov–Smirnov test has been performed to test data distribution. Using the Kruskal–Wallis test, we have analyzed the variables not normally distributed. All analyses have been conducted using online calculators on the https://www.socscistatistics.com website. The analysis of genotypes distribution concordance with Hardy–Weinberg equilibrium and calculations of odds ratios (OR) with confidence intervals (CI) have been performed using the online calculator http://ihg.gsf.de/cgi-bin/hw/hwa1.pl. *P *values below 0.05 were considered to indicate statistical significance.

## Results

All subjected patients (104) showed symptoms of probable awake bruxism in the anamnesis and dental examination. The majority of the studied group constituted women (75%) and young individuals (mean age 29.8 ± 6.5). The pathological attrition of teeth in the anterior segment has occurred in 79 subjected. We have observed that 44 patients (42.3%) revealed a significant attrition of occlusal nodules of posterior teeth. In 82 patients (78.8%), we noticed the formation of enamel scratches, while in 70 subjects (67.3%), we noticed impressions on the tongue.

In addition to physical examination, all patients underwent psychological testing. That has been carried out to measure the level of perceived stress. All 72 patients (69.2%) were qualified as perceiving high-stress levels (between 7 and 9 stens) and 32 (31.8%)—average stress level (6–7 stens). No one patient exhibited stress to a low degree. The relationship between perceived stress level and presence of bruxism symptoms were statistically significant; we observed a moderately strong correlation of stress level and pathological teeth attrition in the anterior segment (*P* < 0.001), enamel attritions (*P* < 0.001), and tongue impressions (*P* < 0.001) and weak correlation with posterior teeth attrition (*P* = 0.005) (Table 1).

**Table 1:** Characteristics of examined patients

Patients characteristics n = 104	Number of patients n (%)	Correlation with stress level in sten scale r, *P* value
Gender		
Men	26 (25.0%)	–
Women	78 (75.0%)	–
Age (mean ± SD)	29.8 ± 6.5	-
Perceived high stress level (7–10 stens)	72 (69.2%)	–
Perceived average stress level (5–6 stens)	32 (30.8%)	–
Perceived low stress level (1–4 stens)	0 (0%)	–
Attrition of anterior teeth	78 (75.0%)	r = 0.45 *P* < 0.001
Attrition of posterior teeth	44 (42.3%)	r = 0.27 *P* = 0.006
Enamel cracks	82 (78.8%)	r = 0.44 *P* < 0.001
Impressions on the tongue	70 (67.3%)	r = 0.50 *P* < 0.001

SD, standard deviation

r, correlation coefficient (point biserial correlation calculation)

Furthermore, we have assumed that bruxism intenseness is related to the number of present symptoms and, for this reason, evaluated the differences in perceived stress level, dividing patients into four groups: with one, two, three, and all four present symptoms. The highest perceived stress level was observed in patients with all four analyzed symptoms (8.0 ± 0.69 stens). Adequately, the lowest perceived stress level was noted in individuals with a single symptom (5.9 ± 0.64 stens). The differences between all groups were statistically significant (*P* < 0.001; data not shown in tables).

Carrying out genotyping, for a start, analyzing c.196G > A (p.Val66Met, rs6265) polymorphism in the *BDNF* gene, we have shown that the c.196A allele (p.74Met) was more frequent in patients with AB than in population control. However, this observation did not achieve considered level of statistical significance (28.4% vs. 22.5%, OR = 1.36, CI = [0.93–2.00], *P* = 0.116, Table 2).

**Table 2:** Alleles and genotypes frequencies for *BDNF* c.196G > A (rs6265) polymorphism in studied groups

	*BDNF* c.196G > A (rs6265)				
	Genotype frequencies (%)			Allele frequencies (%)	
	GG n (%)	GA n (%)	AA n (%)	G n (%)	A n (%)
Patients with bruxism (AB) n = 104	47 (45.1%)	55 (52.9%)	2 (2.0%)	146 (71.6%)	58 (28.4%)
Males (n = 26)	16 (61.5%)	9 (34.6%)	1 (3.9%)	41 (78.9%)	11 (21.1%)
Females (n = 78)	31 (39.7%)	46 (59.0%)	1 (1.3%)	108 (69.2%)	48 (30.8%)
Control group (C) (n = 193)	116 (60.2%)	67 (34.7%)	10 (5.1%)	299 (77.5%) *1000Genomes: 80.0%	87 (22.5%) *1000Genomes: 20.0%
Control males (n = 97)	63 (65.0%)	29 (29.9%)	5 (5.1%)	155 (79.9%)	39 (20.1%)
Control females (n = 96)	53 (55.2%)	38 (39.6%)	5 (5.2%)	144 (75.0%)	48 (25.0%)
*Comparisons of allelic and genotypic frequencies between groups under the study*					
	[GG + GA]vs[AA]	[GG]vs[GA + AA]	[GG]vs[AA]	[G]vs[A]	[A]vs[G]
AB** versus C OR, 95% CI *P *value	OR = 0.36 [0.08–1.67] *P* = 0.174	OR = 0.55 [0.34–0.89] *P* = **0.014**	OR = 2.03 [0.43–9.60] *P* = 0.365	OR = 0.735 [0.50–1.08] *P* = 0.116	OR = 1.36 [0.93–2.00] *P* = 0.116
AB males versus C males OR, 95% CI *P *value	OR = 0.74 [0.08–6.59] *P* = 0.783	OR = 0.86 [0.35–2.11] *P* = 0.747	OR = 1.27 [0.14–11.65] *P* = 0.832	OR = 0.94 [0.44–1.99] *P* = 0.867	OR = 1.07 [0.50–2.26] *P* = 0.867
AB females** versus C females OR, 95% CI *P *value	OR = 0.27 [0.03–2.07] *P* = 0.158	OR = 0.54 [0.29–0.98] *P* = **0.042**	OR = 2.93 [0.33–26.19] *P* = 0.317	OR = 0.75 [0.47–1.20] *P* = 0.231	OR = 1.33 [0.83–2.14] *P* = 0.231
AB females** versus AB males OR, 95% CI *P *value	OR = 0.33 [0.02–5.38] *P* = 0.410	OR = 0.41 [0.17–1.03] *P* = **0.053**	OR = 1.93 [0.11–33.05] *P* = 0.642	OR = 0.60 [0.29–1.28] *P* = 0.183	OR = 1.66 [0.79–3.50] *P* = 0.183
C females versus C males OR, 95% CI *P *value	OR = 1.01 [0.28–3.61] *P* = 0.987	OR = 0.67 [0.37–1.19] *P* = 0.167	OR = 0.84 [0.23–3.06] *P* = 0.793	OR = 0.76 [0.47–1.22] *P* = 0.250	OR = 1.33 [0.82–2.14] *P* = 0.250

In bold were marked statistically significant and borderline significant results (*P* < 0.05). No corrections for the multiple statistical testing were made

[GG + GA] versus [AA] – dominant model, risk allele: G; [GG] versus [GA + AA] – recessive model, risk allele: A

^*^Allele frequencies for the European population according to the 1000Genomes Project have been gathered from http://grch37.ensembl.org/Homo_sapiens/Variation/Population?r=11:27679416-27680416;v=rs6265;vdb=variation;vf=6008 website

^**^HWE analysis revealed discordance in females with AB and a whole group of patients with AB

Considering genotypes distribution, we have observed deviations with Hardy–Weinberg equilibrium (HWE) in the patients’ group. Homozygous genotype GG occurred with lower frequency in patients than in controls (45.1% vs 60.2%). In contrast, heterozygotes GA appeared overrepresented in patients with AB (52.9% vs. 34.7%), and this was statistically relevant ([GG] vs [GA]: OR = 0.49, CI = [0.30–0.81], *P* = 0.005, data not shown in tables). Assuming dominant model with risk allele G the odds ratio was 0.55 (CI = [0.34–0.89], *P* = 0.014). Regarding results and taking into account gender, we pointed out that woman with AB (who constituted the majority in the study group of patients: 75%) showed a lower frequency of homozygotes GG than women from the control group and men with AB as well (39.7% vs 55.2% and 61.5%, respectively). In this comparisons for dominant model and risk allele G the calculated odds ratios were 0.54 (CI = [0.29–0.98], *P* = 0.042) and 0.41 (CI = [0.17–1.03], *P* = 0.053) respectively (Table 2). In female patients with AB, we also observed divergence from the HWE principle. We concluded that the c.196G allele in homozygotes might act as the protective allele, while c.196A of the *BDNF* gene may be associated with AB increased susceptibility.

Going into *NTRK2* gene polymorphism c.1397-31392G > A, we pointed out the higher frequency of the c.1397-31392G allele in the patients than in the control group (55.7% vs 47.4%). Nevertheless this observation was borderline significant (OR = 1.40, CI = [1.00–1.97], *P* = 0.052, Table 3). Homozygous genotype GG occurred more frequently in patients affected with AB than in studied controls (31.4% vs. 21.5%, OR = 1.99, CI = [1.00–3.98]) with *P* = 0.050. Assuming the dominant model with risk allele G, we calculated the odds ratio, which was 1.63 (CI = [0.95–2.79]); however, it was borderline significant (*P* = 0.077) (Table 3).

**Table 3:** Alleles and genotypes frequencies for *NTRK2* c.1397-31392G > A (rs1867283) polymorphism in studied groups

	*NTRK2* c.1397-31392G > A (rs1867283)				
	Genotype frequencies (%)			Allele frequencies (%)	
	GG n (%)	GA n (%)	AA n (%)	G n (%)	A n (%)
Patients with AB n = 104	32 (31.4%)	52 (49.0%)	20 (19.6%)	116 (55.7%)	92 (44.2%)
Males (n = 26)	10 (38.5%)	10 (38.5%)	6 (23.0%)	30 (57.7%)	22 (42.3%)
Females (n = 78)	22 (28.2%)	42 (53.8%)	14 (18.0%)	86 (55.1%)	70 (44.9%)
Control group (C) (n = 191)	41 (21,5%)	99 (51,8%)	51 (26,7%)	181 (47.4%) *1000Genomes: 50.0%	201 (52.6%) *1000Genomes: 50.0%
Control males (n = 96)	24 (25%)	42 (44%)	30 (31%)	90 (46.9%)	102 (53.1%)
Control females (n = 95)	17 (17.9%)	57 (60%)	21 (22.1%)	91 (47.9%)	99 (52.1%)
*Comparisons of allelic and genotypic frequencies between groups under the study*					
	[GG + GA]vs[AA]	[GG]vs[GA + AA]	[GG]vs[AA]	[G]vs[A]	[A]vs[G]
AB versus C OR, 95% CI *P *value	OR = 0.65 [0.37–1.17] *P* = 0.152	OR = 1.63 [0.95–2.79] *P* = 0.077	OR = 1.99 [1.00–3.98] *P* = **0.050**	OR = 1.40 [1.00–1.97]	OR = 0.71 [0.51–1.00]
				*P* = **0.052**	
AB males versus C males OR, 95% CI *P *value	OR = 0.66 [2.41–1.81] *P* = 0.417	OR = 1.86 [0.75–4.68] *P* = 0.175	OR = 2.08 [0.66–6.55] *P* = 0.204	OR = 1.55 [0.83–2.87]	OR = 0.65 [0.35–1.20]
				*P* = 0.166	
AB females** versus C females OR, 95% CI *P *value	OR = 0.77 [0.36–1.64] *P* = 0.498	OR = 1.80 [0.88–3.70] *P* = 0.106	OR = 1.94 [0.77–4.90] *P* = 0.159	OR = 1.34 [0.87–2.04]	OR = 0.75 [0.49–1.14]
				*P* = 0.181	
AB females** versus AB males OR, 95% CI *P *value	OR = 0.73 [0.25–2.15] *P* = 0.566	OR = 0.63 [0.25–1.60] *P* = 0.326	OR = 0.94 [0.28–3.17] *P* = 0.924	OR = 0.90 [0.48–1.70]	OR = 1.11 [0.59–2.09]
				*P* = 0.747	
C females versus C males OR, 95% CI *P *value	OR = 1.01 [0.28–3.61] *P* = 0.987	OR = 0.67 [0.37–1.19] *P* = 0.167	OR = 0.84 [0.23–3.06] *P* = 0.793	OR = 0.76 [0.47–1.22]	OR = 1.33 [0.82–2.14]
				*P* = 0.215	

In bold were marked borderline statistically significant results (*P*-value < 0.05). No corrections for the multiple statistical testing were made

[GG + GA] versus [AA]—dominant model, risk allele: G

[GG] versus [GA + AA]—recessive model, risk allele: A

^*^Allele frequencies for the European population according to the 1000Genomes Project have been gathered from http://grch37.ensembl.org/Homo_sapiens/Variation/Population?db=core;r=9:87450266-87451266;v=rs1867283;vdb=variation;vf=1303363 website

^**^HWE analysis revealed discordance in the group of females with AB

Carrying out comparisons according to gender, we have observed the highest rate of homozygotes GG among men with AB: 38.5% compared to 28.2% in women with AB and 25% in control males, and 17.9% in control females. Although we have not demonstrated statistically evidenced differences between genders and groups under the study in genotypes distribution, the GG genotype was overrepresented in male patients with AB against control males (OR = 1.86) and similarly in female patients against women from the control group (OR = 1.80), (Table 3). In rs1867283 locus, we also observed the deviation from HWE in women with AB.

In the following step, we have assessed the relationship of genotypes and alleles of analyzed loci with clinical symptoms in the studied group of patients (Table 4).

**Table 4:** *BDNF* c.196G > A and *NTRK2* c.1397-31392G > A alleles association and correlation of genotypes with AB and clinical presentation

Parameter	*BDNF* c.196G > A (rs6265)							*NTRK2* c.1397-31392G > A (rs1867283)						
								n = 104						
	GG (n = 47)	GA (n = 55)	AA (n = 2)	[GG + GA] versus [AA] OR, CI *P *value	[GG] versus [GA + AA] OR, CI *P *value	[G] versus [A] OR, CI	[A] versus [G] OR, CI	GG (n = 32)	GA (n = 52)	AA (n = 20)	[GG + GA] versus [AA] OR, CI *P *value	[GG] versus [GA + AA] OR, CI *P *value	[G] versus [A] OR, CI	[A] versus [G] OR, CI
						*P *value							*P *value	
The perceived stress level in sten scale (mean ± SD)	7.13 ± 1.15	7.11 ± 1.10	7.50 ± 2.12	NA	NA	NA	NA	7.22 ± 1.10	7.10 ± 1.16	7.25 ± 1.16	NA	NA	NA	NA
	*P* = 0.840							*P* = 0.629						
Attrition of anterior teeth n = 78	40 (51.3%)	38 (48.7)	0 (0%)	OR = 1.40 CI = [0.07–30.17] *P* = 0.460	OR = 2.86 CI = [1.08–7.57] *P* = **0.031**	OR = 2.10 CI = [1.08–4.09]	OR = 0.48 CI = [0.25–0.92]	26 (33.3%)	38 (48.7%)	14 (18%)	OR = 0.73 CI = [0.25–2.15] *P* = 0.566	OR = 1.67 CI = [0.60–4.66] *P* = 0.326	OR = 1.36 CI = [0.73–2.56]	OR = 0.73 CI = [0.39–1.38]
						*P* = **0.026**							*P* = 0.333	
Attrition of posterior teeth n = 44	28 (63.6%)	16 (36.4%)	0 (0%)	OR = 0.26 CI = [0.01–5.62] *P* = 0.221	OR = 3.78 CI = [1.66–8.58] *P* = **0.001**	OR = 2.51 CI = [1.30–4.85]	OR = 0.40 CI = [0.21–0.77]	14 (31.8%)	26 (59.1%)	4 (9.1%)	OR = 0.28 CI = [0.09–0.89] *P* = **0.025**	OR = 1.09 CI = [0.47–2.53] *P* = 0.843	OR = 1.49 CI = [0.85–2.60]	OR = 0.67 CI = [0.39–1.18]
						*P* = **0.005**							*P* = 0.164	
Enamel cracks n = 82	43 (52.5%)	37 (45.1%)	2 (2.4%)	OR = 1.40 CI = [0.07–30.17] *P* = 0.460	OR = 4.96 CI = [1.55–15.94] *P* = **0.004**	OR = 2.08 CI = [1.03–4.17]	OR = 0.48 CI = [0.24–0.97]	22 (26.8%)	44 (53.7%)	16 (19.5%)	OR = 1.09 CI = [0.32–3.67] *P* = 0.888	OR = 0.44 CI = [0.17–1.16] *P* = 0.093	OR = 0.66 CI = [0.33–1.32]	OR = 1.51 CI = [0.76–3.00]
						*P* = **0.038**							*P* = 0.237	
Impressions on the tongue n = 70	33 (47.2%)	36 (51.4%)	1 (1.4%)	OR = 0.48 CI = [0.03–7.89] *P* = 0.598	OR = 1.27 CI = [0.56–2.92] *P* = 0.566	OR = 1.20 CI = [0.64–2.26]	OR = 0.83 CI = [0.44–1.57]	16 (22.9%)	38 (54.2%)	16 (22.9%)	OR = 2.22 CI = [0.68–7.25] *P* = 0.178	OR = 0.33 CI = [0.14–0.80] *P* = **0.012**	OR = 0.48 CI = [0.26–0.88]	OR = 2.09 CI = [1.14–3.84]
						*P* = 0.575							*P* = **0.016**	

In bold were marked statistically significant and borderline significant results (*P* < 0.05). No corrections for the multiple statistical testing were made

[GG + GA] versus [AA]—dominant model, risk allele: G; [GG] versus [GA + AA]—recessive model, risk allele: A

Regarding *BDNF* c.196G > A variant, surprisingly, we observed that assessed clinical symptoms in a study group were associated rather with the c.196G allele. These observations were statistically significant in case of attrition of anterior as well as posterior teeth (OR = 2.10, *P* = 0.026 and OR = 2.51, *P* = 0.005, respectively) and enamel cracks (OR = 2.08, *P* = 0.038) (Table 4).

Overall, the c.196A allele may act as a susceptibility allele, but it is not associated with clinical characteristics evaluated in patients assessed in this study.

The analysis of *NTRK2* c.1397-31392G > A polymorphism relationship with clinical course showed that this is allele c.1397-31392A, which is associated with the presence of tongue impressions (OR = 2.09, *P* = 0.016), and GG genotype is protective (OR = 0.33, CI = [0.14–0.80], *P* = 0.012) (Table 4).

## Discussion

Our results demonstrated that perceived stress level is a substantial contributing factor to the presence of awake bruxism and its clinical manifestations. Moreover, polymorphic variants in genes related to stress coping: c.196G > A (p.Val66Met, rs6265) in the *BDNF* gene and c.1397-31392G > A in the *NTRK2* (rs1867283) gene may also be related to the AB susceptibility and clinical presentation. Our results should be interpreted as preliminary, given that the study group was not numerous and only single SNPs were assessed. Nonetheless, they contribute to the understanding of the molecular basis of bruxism.

It was shown earlier that the c.196G > A nonsynonymous substitution in the *BDNF* gene influence *BDNF* mRNA localization, alter the intracellular distribution and activity-dependent secretion of the BDNF protein affecting signal transmission at the neuronal level. While the c.196G > A substitution in the *BDNF* gene has a straightforward consequence on the protein level, resulting in amino acid change (p.Val66Met) affecting the neurotrophin activity [24], the c.1397-31392G > A variation in the *NTRK2* gene is located in an intron, and their effect on gene function remains unknown. Still, it may affect the neurotrophin receptor level straightway or be in linkage disequilibrium with another gene sequence variant directly affecting neurotrophin receptor level or activity.

The literature presents peripheral and central sources of the bruxism phenomenon. Peripheral factors include genetic and psychological factors, such as stress and fear [11]. Manfredini et al. suggested that bruxism and other stomatognathic system parafunction have a central aetiology rather. In this context, the role of the psychological component assumes great importance, mainly because of its relationship to the central nervous system (CNS), which regulates emotions [25]. Wieckiewicz et al. suggested that bruxism occurring during the night is a complex condition resulting from multiple interactions between genetic and environmental factors. They also underlined the indirect influence of phenotypes such as obesity, craniofacial structure, neurological control of upper airway muscles and sleep, and circadian rhythm [14].

Nonetheless, all central and peripheral factors work together and depend on each other. Chronic stress is linked closely to the abnormal functions of the masticatory organ, which differ qualitatively and quantitatively from the physiological pattern. These atypical features arise to discharge emotional tension, causing excessive contraction of muscle groups within the US, resulting in a long-lasting and non-physiological strain on tissues [26].

Neurotrophin has been shown to have antidepressant properties, and several antidepressants modulate the receptor tyrosine kinase B (TrkB) signalling in a BDNF-dependent manner in the cerebral cortex [27]. Moreover, chronic stress induces hippocampal atrophy and reduces the *BDNF* gene expression in limbic structures that take part in mood regulation, including the hippocampus and prefrontal cortex [28]. Aizawa et al. observed a relationship between *BDNF* gene variant c.196G > A and emotion-focused strategies, seeking social support, self-control, distancing, and critical attitudes. They reported that individuals with the c.196A allele displayed more signs of anxiety and scored higher in the stress-coping ability than those carrying the c.196G allele. Moreover, in their studies, carriers of the c.196A allele had lower scores in the ego aptitude scale, and they may have a weakened ego, while individuals with higher critical attitude scores tend to have more physical and mental health issues [19]. In this light, our results are inconclusive: the c.196A allele was slightly more frequent in AB patients, nonetheless analyzing the presence of clinical symptoms, we revealed the relationship of attrition of anterior and posterior teeth and enamel cracks with c.196G allele and GG genotype.

NTRK2 is a specific TrkB receptor for BDNF and has a regulatory role in neural differentiation and the maintenance of neural cells in the human prefrontal cortex [29]. Polymorphisms in the *NTRK2* gene increase risk of architectural changes in several brain regions involved in emotional regulation. Aizawa et al. found significant associations between polymorphisms in this gene and cognitive strategies, problem-solving, seeking social support, distancing, positive reappraisal. They associated homozygous minor genotype with a significantly lower score and attenuated stress coping style than other genotypes [19], which is in concordance with our results and indicates a possible relationship between *NTRK2* gene sequence variants and susceptibility to AB.

Moreover, Oporto et al. reported that polymorphisms in serotonergic pathways are involved in sleep bruxism [30]. The same group in the latest report suggested the role of genetic polymorphisms of dopaminergic pathway genes in circadian manifestations of bruxism [31]. The latest study of Wieckiewicz et al. linked the prevalence of sleep bruxism with serotonin and dopamine pathways. Their findings revealed the relationship of the sequence variants within the dopamine receptor gene with the etiology of sleep bruxism. They also revealed the possible impact of the polymorphisms in the serotonin receptor encoding gene on the association between sleep bruxism and obstructive sleep apnea [14].

Bruxism is significant but still underestimated epidemiological issue. It is still a controversial and often-discussed topic in dentistry because dentists do not have enough standardized tools to assess Bruxism (STAB). According to Manfredini et al., an accurate estimation of bruxism is problematic due to different diagnostic strategies, non-representative populations, and comorbid conditions that may act as confounding variables [25]. Additionally, dentally-based diagnosis of treatment and prevention demanding bruxism is not accurate in the absence of control for other potential causes of tooth wear (e.g., functional, endogenous, or exogenous factors). The final goal of providing STAB is to enable and facilitate easy comparison of studies in the research setting and, what is most important, provide better patient care in the clinical setting. Nowadays, this is extremely important in the COVID-19 pandemic reality because it is widely observed in clinical practices and even shown in the latest reports that the number of bruxism and temporomandibular disorders in those suffering from a compromised psychoemotional state increase significantly [32].

Our study has several limitations. First, the clinical diagnostic criteria may be improved; we could not diagnose definite awake bruxism. Moreover, we did not perform polysomnography and could not exclude the coexistence of SB. Secondly, the study group was not numerous and with gender imbalance. Also, we studied only two SNPs. However, this report may be treated as preliminary and replicated in more numerous cohorts and different populations.

## Conclusions

Hence, our results suggest that awake bruxism is a complex disease with multigene conditioning with an additive effect of several genetic and environmental factors. Sequence variants of genes related to stress coping may be associated with the pathogenesis of awake bruxism and should be investigated in more numerous groups and different populations. Identifying genetic markers of higher susceptibility becomes one of the crucial in bruxism diagnosis. Moreover, a better understanding of molecular basis allows developing treatment possibilities in the short future and will enable effective treatment schemes.

## Data Availability

The datasets generated during the current study are available from the corresponding author on reasonable request.

## Abbreviations

AB: Awake bruxism
SB: Sleep bruxism
BDNF: Brain-derived neurotrophic factor, neurotrophin
NTRK: Neurotrophin receptor
TrkB: Tyrosine kinase B
GWAS: Genome-wide association studies
OBC: Oral behaviours checklist
PSS: Perceived Stress Scale
HRMA: High resolution melting analysis
HWE: Hardy–Weinberg equilibrium
SD: Standard deviation
r: Correlation coefficient
OR: Odds ratio
STAB: Standardized tools to assess bruxism
